# Crystal structure and Hirshfield surface analysis of 4-phenyl-3-(thio­phen-3-ylmeth­yl)-1*H*-1,2,4-triazole-5(4*H*)-thione

**DOI:** 10.1107/S2056989018007193

**Published:** 2018-05-18

**Authors:** Trung Vu Quoc, Linh Nguyen Ngoc, Dai Do Ba, Thang Pham Chien, Hung Nguyen Huy, Luc Van Meervelt

**Affiliations:** a Faculty of Chemistry, Hanoi National University of Education, 136 Xuan Thuy, Cau Giay, Hanoi, Vietnam; b Department of Chemistry, Hanoi University of Science, 19 Le Thanh Tong Street, Ha Ba Discrict, Hanoi, Vietnam; c Department of Chemistry, KU Leuven, Biomolecular Architecture, Celestijnenlaan 200F, Leuven (Heverlee), B-3001, Belgium

**Keywords:** crystal structure, thio­phene, 1,2,4-triazole, thione tautomer, Hirshfield surfaces

## Abstract

In the title compound, C_13_H_11_N_3_S_2_, the phenyl ring is twisted from the 1,2,4-triazole plane by 63.35 (9)° and by 47.35 (9)° from the thio­phene plane. In the crystal, chains of mol­ecules running along the *c*-axis direction are formed by N—H⋯S inter­actions [graph-set motif *C*(4)]. The 1,2,4-triazole and phenyl rings are involved in π–π stacking inter­actions [centroid–centroid distance = 3.4553 (10) Å]. The thio­phene ring is involved in C—H⋯S and C—H⋯π inter­actions. The inter­molecular inter­actions in the crystal packing were further analysed using Hirshfield surface analysis, which indicates that the most significant contacts are H⋯H (35.8%), followed by S⋯H/H⋯S (26.7%) and C⋯H/H⋯C (18.2%).

## Abstract 2

The synthesis and crystal structure of a new thio­phene monomer containing a 1,2,4-triazole-5-thione ring are reported. The thio­phene and 1,2,4-triazole rings are inclined to each other by 79.70 (9)°.

## Chemical context   

The triazole ring is an important component of bioactive heterocycles because of its effect in bactericides, pesticides and fungicides (Sengupta *et al.*, 1978 ▸; Singh *et al.*, 1979 ▸; Giri *et al.*, 1978 ▸). Many derivatives containing 1,2,4-triazoline-5-thione show a variety of biological activities: anti-inflamatory (Sahin *et al.*, 2001 ▸), anti­fungal (Knight *et al.*, 1978 ▸, 1979 ▸), analgesis (Mekuskiene *et al.*, 1998 ▸) and bacteriostatic (Eweiss *et al.*, 1986 ▸; Mazzone *et al.*, 1981 ▸). Thio­phene-containing 1,2,4-triazole derivatives have been studied and these compounds have shown promising anti­mycotic activity (Wujec *et al.*, 2004 ▸). Combinations of the thio­phene ring with other heterocyclic rings applied in conducting polymers have also been investigated (Ho *et al.*, 2002 ▸; Mohamed *et al.*, 2014 ▸; Bondarev *et al.*, 2010 ▸).
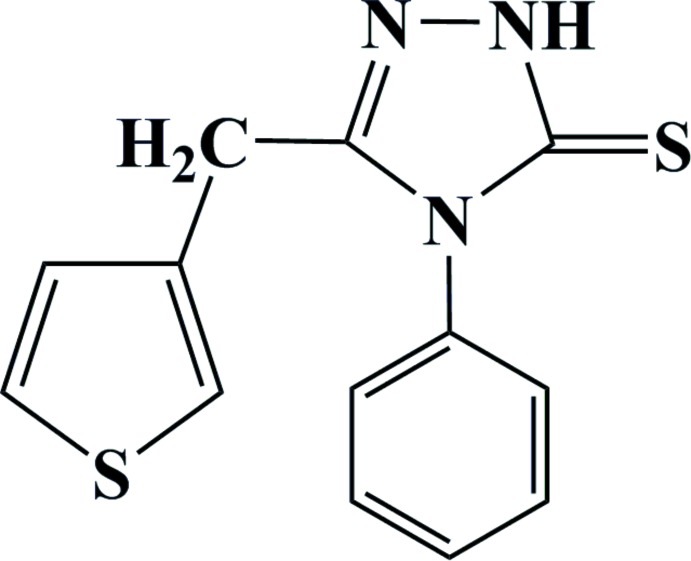


As part of our studies, we have synthesized a new thio­phene monomer containing 1,2,4-triazole-5-thione. The polymer obtained from 4-phenyl-3-(thio­phen-3-yl-meth­yl)-1*H*-1,2,4-triazole-5(4*H*)-thione was further characterized by IR spectroscopy and TGA. TG–TGA analysis shows that the polymer is thermally stable above 473 K, showing degradation beyond 773 K and exothermic maxima at 745 K. We present here the synthesis and crystal structure of the title compound.

## Structural commentary   

**Figure 1:**
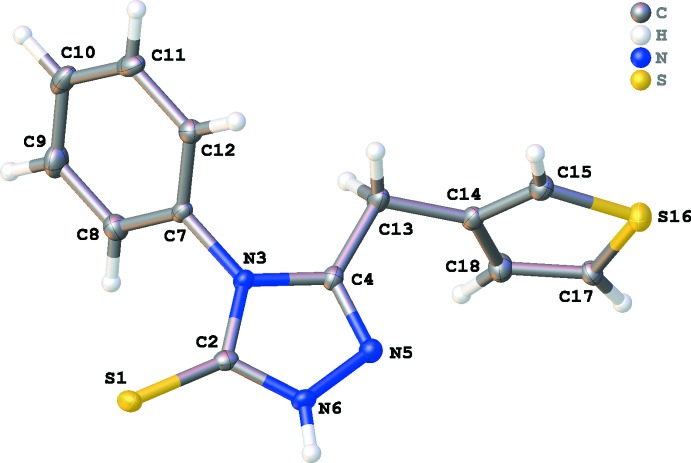
View of the asymmetric unit of the title compound, showing the atom-labelling scheme. Displacement ellipsoids are drawn at the 50% probability level. H atoms are shown as small circles of arbitrary radii.

The title compound crystallizes in the monoclinic space group *P2_1_/c* with one mol­ecule in the asymmetric unit (Fig. 1 ▸). In the crystalline state, the central 1,2,4-triazole ring exists in its thione tautomeric state with a C2=S1 distance of 1.6845 (16) Å. The short C4=N5 distance [1.302 (2) Å] indicates its double-bond character. The 1,2,4-triazole ring is almost planar (r.m.s. deviation = 0.002 Å for ring C2/N3/C4/N5/N6), with the substituents S1, C7 and C13 deviating by −0.020 (1), −0.028 (2) and 0.061 (2) Å, respectively. The plane of the 1,2,4-triazole ring forms dihedral angles of 79.70 (9) and 63.35 (9)° with the best planes through the thio­phene and phenyl rings, respectively. The thio­phene and phenyl rings are inclined to each other by 47.35 (9)°. The thio­phene ring does not show rotational disorder as observed in previous structure determinations of similar compounds (Vu Quoc *et al.*, 2017 ▸).

## Supra­molecular features   

**Figure 2:**
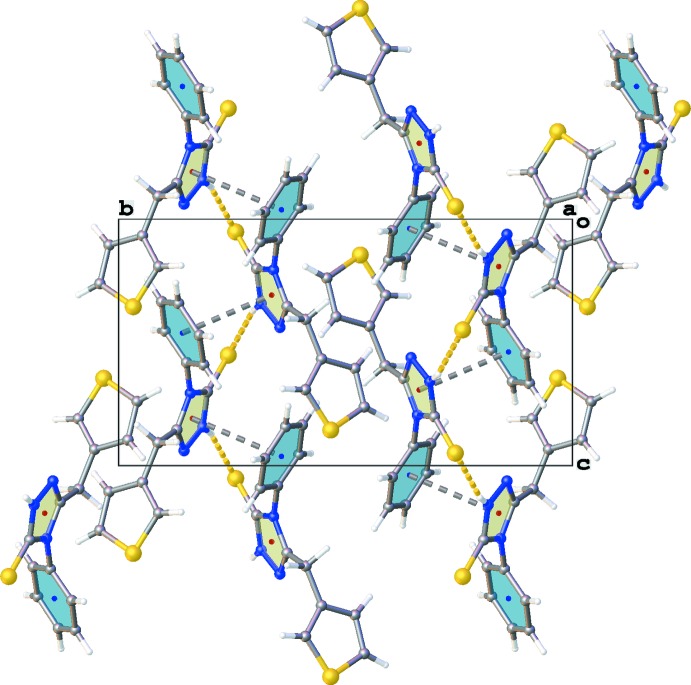
Crystal packing of the title compound shown in projection down the *a* axis illustrating chain formation along the *c*-axis direction by N—H⋯S hydrogen bonding (yellow dashed lines) and the π–π stacking inter­actions between the 1,2,4-triazole (yellow) and phenyl (blue) rings.

The crystal packing of the title compound is shown in Fig. 2 ▸. The packing is dominated by N6—H6⋯S1 inter­actions (Table 1 ▸), resulting in the formation of chains of mol­ecules with graph-set motif *C*(4) propagating along the *c*-axis direction. In addition, the 1,2,4-triazole and phenyl rings exhibit π–π stacking inter­actions [*Cg*2⋯*Cg*3^i^ = 3.4553 (10) Å; angle of inclination = 9.98 (9)°; *Cg*2 and *Cg*3 are the centroids of the 1,2,4-triazole and phenyl rings, respectively; symmetry code: (i) *x*, −*y* + 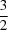, *z* + 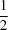; Fig. 2 ▸].

**Figure 3:**
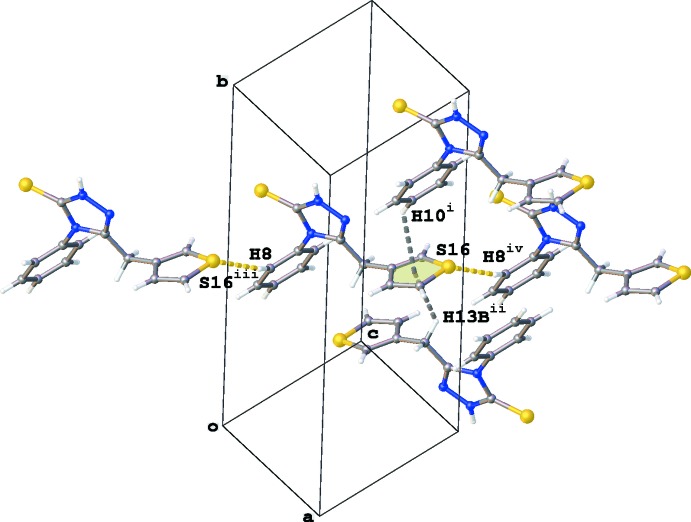
Partial crystal packing of the title compound, showing the C—H⋯π (gray dashed lines) and C—H⋯S inter­actions (yellow dashed lines) [see Table 1 ▸; symmetry codes: (i) *x*, *y*, *z* + 1; (ii) −*x* + 2, −*y* + 1, −*z* + 1; (iii) *x* − 1, *y*, *z* − 1; (iv) *x* + 1, *y*, *z* + 1].

The thio­phene ring plays also a role in the crystal packing as illustrated by the weaker C8—H8⋯S16 inter­actions and C—H⋯π inter­actions involving H atoms H10 and H13*B* (Table 1 ▸, Fig. 3 ▸). The crystal packing contains no voids.

## Hirshfield surface analysis   

**Figure 4:**
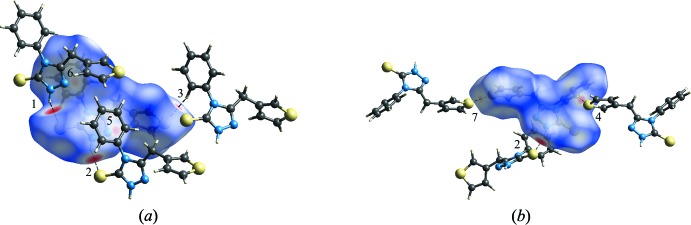
Two views of the Hirshfield surface for the title compound mapped over *d*_norm_ in the range −0.386 to +1.111 a.u., showing (*a*) the N—H⋯S and C—H⋯S hydrogen bonding and π–π inter­actions between the 1,2,4-triazole and phenyl rings, and (*b*) the N—H⋯S and C—H⋯S hydrogen bonding and S⋯S inter­actions.

Hirshfield surface and two-dimensional fingerprint plot calculations were performed using *CrystalExplorer* (McKinnon *et al.*, 2007 ▸; Spackman & Jayatilaka, 2009 ▸). The larger bright-red spots near atoms S1, N6, S16 and H8 (labelled 1, 2, 3 and 4) on the Hirshfield surface mapped over *d*_norm_ in Fig. 4 ▸ *a* and *b* represent the N—H⋯S and C—H⋯S hydrogen bonds present in the crystal packing. The pale-red spots in Fig. 4 ▸ *a* near atom N5 and the phenyl ring (labelled 5 and 6) are the result of the π–π stacking between the 1,2,4-triazole and phenyl rings. In Fig. 4 ▸ *b*, an additional pale-red spot is present near atom S16 (labelled 7), indicating a short S⋯S contact [S16⋯S16^i^ = 3.4688 (7) Å; symmetry code: (i) −*x* + 2, −*y* + 1, −*z* + 2]. The relative contributions of the different inter­molecular inter­actions to the Hirshfield surface area, in descending order, are: H⋯H (35.8%), S⋯H/H⋯S (26.7%), C⋯H/H⋯C (18.2%), N⋯H/H⋯N (8.5%), C⋯N/N⋯C (3.7%), C⋯C (3.1%), S⋯C/C⋯S (2.8%) and S⋯S (1.2%). The latter value indicates that the S⋯S contact only makes a marginal contribution to the packing of the title compound.

## Database survey   

A search of the Cambridge Structural Database (CSD, Version 5.39, last update November 2017; Groom *et al.*, 2016 ▸) for crystal structures containing a 1*H*-1,2,4-triazole-5(4*H*)-thione moiety results in 287 (only organics) or 375 structures (including organometallics). When considering only organics, the average C=S and C=N distances are, respectively, 1.676 (9) Å [ranging from 1.608 to 1.699 Å] and 1.302 (11) Å [ranging from 1.275 to 1.410 Å]. For the 66 structures with atom N3 bearing a phenyl subsituent (only organics), the dihedral angle between the 1,2,4-triazole and phenyl rings varies from 55.3 to 90° (the latter when bulky substituents are present at position C4). In the case of a –CH_2_ *R* group at position C4, 53 structures are retrieved from the CSD. In this case, the torsion angle N=C—CH_2_—*R* shows three favoured regions: (1) *synperiplanar* for small subsituents (torsion angles between −23 and +32°, 28 hits), (2) *+anti­clinal* (torsion angles between 67 and 115°, 15 hits) and (3) −*anti­clinal* (torsion angles between −87 and −140°, 10 hits).

## Synthesis and crystallization   

**Figure 5:**
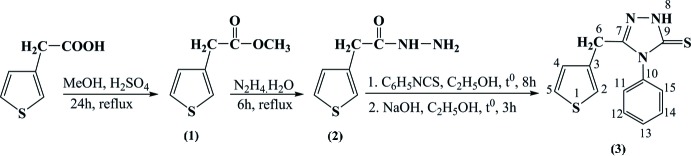
Reaction scheme for the title compound.

The reaction scheme used to synthesize the title compound, **(3)**, is given in Fig. 5 ▸. Methyl 2-(thio­phen-3-yl)acetate, **(1)**, and 2-(thio­phen-3-yl)acetohydrazide, **(2)**, were synthesized as described in a previous study (Vu Quoc *et al.*, 2017 ▸).

A mixture of hydrazide **(2)** (0.01 mol), phenyl­iso­thio­cyanate (0.01 mol) and 20 mL ethanol was refluxed at 353 K for 8h. The solid precipitate was filtered, washed and recrystallized from ethanol to give white crystals (m.p. 416 K). Then, the mixture of the resulting solid (0.411 g), 10 mL ethanol and NaOH 10% (1.25 mmol) was refluxed at 353 K for 3 h. The reaction mixture was cooled and neutralized with HCl 10% to pH = 1–2. The product was filtered, washed and recrystallized from ethanol to give 1.42 g (yield 52%) of **(3)** in the form of pale-yellow crystals (m.p. 451 K). IR (Nicolet Impact 410 FTIR, KBr, cm^−1^): 3453 (NH), 3088, 2911 (CH), 1576 (C=C thio­phene), 1278, 1207 (C=S). ^1^H NMR [Bruker XL-500, 500 MHz, *d*_6_-DMSO, δ (ppm), *J* (Hz)]: 6.96 (*m*, 1H, H^2^), 6.75 (*d*, 1H, ^5^ *J* = 4.5, H^4^), 7.38 (*dd*, 1H, ^2^ *J* = 3.0, ^4^ *J* = 5.0, H^5^), 3.85 (*s*, 2H, H^6^), 13.77 (*s*, 1H, H^8^), 7.26–7.28 (*m*, 2H, H^11^ and H^15^), 7.48–7.50 (*m*, 3H, H^12^, H^13^ and H^14^). ^13^C NMR [Bruker XL-500, 125 MHz, *d*_6_-DMSO, δ (ppm)]: 123.86 (C2), 134.24 (C3), 128.02 (C4), 126.14 (C5), 26.35 (C6), 150.83 (C7), 167.85 (C9), 133.55 (C10), 128.16 (C11 and C15), 129.20 (C12 and C14), 129.34 (C13). Calculation for C_13_H_11_N_3_S_2_: *M* = 273 a.u.

## Refinement   

Crystal data, data collection and structure refinement details are summarized in Table 2 ▸. The H atoms were placed at calculated positions and refined in riding mode, with a N—H distance of 0.88 Å or C—H distances of 0.95 (aromatic) and 0.99 Å (CH_2_), and isotropic displacement parameters equal to 1.2*U*_eq_ of the parent atoms.

## Supplementary Material

Crystal structure: contains datablock(s) I. DOI: 10.1107/S2056989018007193/zp2029sup1.cif

Structure factors: contains datablock(s) I. DOI: 10.1107/S2056989018007193/zp2029Isup2.hkl

Supporting information file. DOI: 10.1107/S2056989018007193/zp2029Isup3.cml

CCDC reference: 1843042

Additional supporting information:  crystallographic information; 3D view; checkCIF report

## Figures and Tables

**Table 1:** Hydrogen-bond geometry (Å, °) *Cg*1 is the centroid of the C14/C15/S16/C17/C18 thio­phene ring.

*D*—H⋯*A*	*D*—H	H⋯*A*	*D*⋯*A*	*D*—H⋯*A*
N6—H6⋯S1^i^	0.88	2.46	3.2866 (16)	156
C8—H8⋯S16^ii^	0.95	2.82	3.737 (2)	162
C10—H10⋯*Cg*1^iii^	0.95	2.83	3.566 (2)	135
C13—H13*B*⋯*Cg*1^iv^	0.99	2.76	3.409 (2)	123

Symmetry codes: (i) 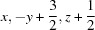; (ii) 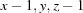; (iii) 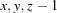; (iv) 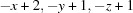.

**Table 2:** Experimental details

Crystal data	
Chemical formula	C_13_H_11_N_3_S_2_
*M*_r_	273.37
Crystal system, space group	Monoclinic, *P*2_1_/*c*
Temperature (K)	100
*a*, *b*, *c* (Å)	8.8257 (8), 16.0776 (16), 9.7437 (9)
β (°)	116.383 (3)
*V* (Å^3^)	1238.6 (2)
*Z*	4
Radiation type	Mo *K*α
μ (mm^−1^)	0.41
Crystal size (mm)	0.31 × 0.21 × 0.09

Data collection	
Diffractometer	Bruker D8 Quest CMOS
Absorption correction	Multi-scan (*SADABS*; Bruker, 2014 ▸)
*T*_min_, *T*_max_	0.700, 0.746
No. of measured, independent and observed [*I* > 2σ(*I*)] reflections	20908, 3082, 2697
*R*_int_	0.038
(sin θ/λ)_max_ (Å^−1^)	0.668

Refinement	
*R*[*F* ^2^ > 2σ(*F* ^2^)], *wR*(*F* ^2^), *S*	0.035, 0.097, 1.06
No. of reflections	3082
No. of parameters	163
H-atom treatment	H-atom parameters constrained
Δρ_max_, Δρ_min_ (e Å^−3^)	0.59, −0.38

Computer programs: *APEX2* (Bruker, 2014 ▸), *SAINT* (Bruker, 2013 ▸), *SHELXT* (Sheldrick, 2015*a* ▸), *SHELXL* (Sheldrick, 2015*b* ▸) and *OLEX2* (Dolomanov *et al.*, 2009 ▸).

## supplementary crystallographic information

### Crystal data

**Table:**

C_13_H_11_N_3_S_2_	*F*(000) = 568
*M**_r_* = 273.37	*D*_x_ = 1.466 Mg m^−^^3^
Monoclinic, *P*2_1_/*c*	Mo *K*α radiation, λ = 0.71073 Å
*a* = 8.8257 (8) Å	Cell parameters from 9914 reflections
*b* = 16.0776 (16) Å	θ = 2.9–28.3°
*c* = 9.7437 (9) Å	µ = 0.41 mm^−^^1^
β = 116.383 (3)°	*T* = 100 K
*V* = 1238.6 (2) Å^3^	Block, yellow
*Z* = 4	0.31 × 0.21 × 0.09 mm

### Data collection

**Table:**

Bruker D8 Quest CMOS diffractometer	2697 reflections with *I* > 2σ(*I*)
φ and ω scans	*R*_int_ = 0.038
Absorption correction: multi-scan (SADABS; Bruker, 2014)	θ_max_ = 28.4°, θ_min_ = 2.9°
*T*_min_ = 0.700, *T*_max_ = 0.746	*h* = −11→11
20908 measured reflections	*k* = −21→21
3082 independent reflections	*l* = −12→12

### Refinement

**Table:**

Refinement on *F*^2^	Primary atom site location: dual
Least-squares matrix: full	Hydrogen site location: inferred from neighbouring sites
*R*[*F*^2^ > 2σ(*F*^2^)] = 0.035	H-atom parameters constrained
*wR*(*F*^2^) = 0.097	*w* = 1/[σ^2^(*F*_o_^2^) + (0.0467*P*)^2^ + 1.0177*P*] where *P* = (*F*_o_^2^ + 2*F*_c_^2^)/3
*S* = 1.06	(Δ/σ)_max_ = 0.001
3082 reflections	Δρ_max_ = 0.59 e Å^−^^3^
163 parameters	Δρ_min_ = −0.38 e Å^−^^3^
0 restraints	

### Special details

**Table:**

Geometry. All esds (except the esd in the dihedral angle between two l.s. planes) are estimated using the full covariance matrix. The cell esds are taken into account individually in the estimation of esds in distances, angles and torsion angles; correlations between esds in cell parameters are only used when they are defined by crystal symmetry. An approximate (isotropic) treatment of cell esds is used for estimating esds involving l.s. planes.

### Fractional atomic coordinates and isotropic or equivalent isotropic displacement parameters (Å^2^)

**Table:**

	*x*	*y*	*z*	*U*_iso_*/*U*_eq_	
S1	0.24329 (5)	0.74179 (3)	0.05131 (5)	0.01795 (11)	
C2	0.40447 (19)	0.69570 (10)	0.19985 (17)	0.0138 (3)	
N3	0.54765 (16)	0.65826 (8)	0.20563 (14)	0.0132 (3)	
C4	0.64077 (19)	0.62860 (10)	0.35345 (17)	0.0141 (3)	
N5	0.56488 (17)	0.64436 (8)	0.43857 (15)	0.0158 (3)	
N6	0.42054 (16)	0.68568 (8)	0.34200 (15)	0.0151 (3)	
H6	0.345269	0.703954	0.371018	0.018*	
C7	0.59244 (19)	0.64996 (9)	0.08099 (17)	0.0130 (3)	
C8	0.4894 (2)	0.60295 (10)	−0.04588 (18)	0.0184 (3)	
H8	0.390920	0.576602	−0.050509	0.022*	
C9	0.5328 (2)	0.59507 (11)	−0.16594 (19)	0.0235 (4)	
H9	0.462216	0.563899	−0.254176	0.028*	
C10	0.6780 (2)	0.63219 (11)	−0.1584 (2)	0.0225 (4)	
H10	0.707029	0.626318	−0.240840	0.027*	
C11	0.7808 (2)	0.67794 (11)	−0.02992 (19)	0.0201 (3)	
H11	0.881215	0.702779	−0.023979	0.024*	
C12	0.7379 (2)	0.68777 (10)	0.09068 (18)	0.0163 (3)	
H12	0.807211	0.719883	0.178022	0.020*	
C13	0.8089 (2)	0.58721 (11)	0.40650 (18)	0.0183 (3)	
H13A	0.891635	0.627925	0.403638	0.022*	
H13B	0.799421	0.541364	0.335289	0.022*	
C14	0.87343 (19)	0.55283 (10)	0.56653 (18)	0.0154 (3)	
C15	1.0020 (2)	0.58833 (10)	0.69147 (18)	0.0174 (3)	
H15	1.058440	0.638022	0.687921	0.021*	
S16	1.04926 (5)	0.53259 (3)	0.85492 (5)	0.02086 (12)	
C17	0.89607 (19)	0.45897 (10)	0.76090 (17)	0.0150 (3)	
H17	0.872539	0.412045	0.807510	0.018*	
C18	0.8116 (2)	0.47912 (11)	0.60493 (19)	0.0196 (3)	
H18	0.721852	0.446760	0.532024	0.024*	

### Atomic displacement parameters (Å^2^)

**Table:**

	*U*^11^	*U*^22^	*U*^33^	*U*^12^	*U*^13^	*U*^23^
S1	0.01244 (19)	0.0249 (2)	0.0173 (2)	0.00407 (14)	0.00726 (15)	0.00491 (15)
C2	0.0135 (7)	0.0139 (7)	0.0161 (7)	−0.0021 (5)	0.0084 (6)	−0.0012 (5)
N3	0.0134 (6)	0.0155 (6)	0.0117 (6)	0.0015 (5)	0.0066 (5)	0.0012 (5)
C4	0.0159 (7)	0.0148 (7)	0.0115 (7)	−0.0002 (5)	0.0061 (6)	0.0009 (5)
N5	0.0159 (6)	0.0175 (6)	0.0146 (6)	0.0017 (5)	0.0073 (5)	0.0016 (5)
N6	0.0147 (6)	0.0178 (6)	0.0154 (6)	0.0019 (5)	0.0091 (5)	0.0001 (5)
C7	0.0141 (7)	0.0147 (7)	0.0120 (6)	0.0038 (5)	0.0073 (5)	0.0024 (5)
C8	0.0163 (7)	0.0208 (8)	0.0164 (7)	0.0002 (6)	0.0058 (6)	−0.0006 (6)
C9	0.0274 (9)	0.0267 (9)	0.0145 (7)	0.0019 (7)	0.0075 (7)	−0.0030 (6)
C10	0.0284 (9)	0.0267 (9)	0.0176 (8)	0.0108 (7)	0.0150 (7)	0.0061 (7)
C11	0.0185 (7)	0.0235 (8)	0.0226 (8)	0.0049 (6)	0.0132 (7)	0.0083 (6)
C12	0.0154 (7)	0.0182 (8)	0.0151 (7)	0.0013 (6)	0.0067 (6)	0.0021 (6)
C13	0.0184 (7)	0.0238 (8)	0.0143 (7)	0.0070 (6)	0.0087 (6)	0.0053 (6)
C14	0.0157 (7)	0.0178 (7)	0.0137 (7)	0.0046 (6)	0.0073 (6)	0.0028 (6)
C15	0.0179 (7)	0.0183 (8)	0.0165 (7)	0.0007 (6)	0.0080 (6)	0.0023 (6)
S16	0.0193 (2)	0.0267 (2)	0.0146 (2)	−0.00099 (16)	0.00573 (16)	0.00087 (15)
C17	0.0129 (7)	0.0168 (7)	0.0132 (7)	−0.0010 (5)	0.0038 (6)	−0.0004 (5)
C18	0.0194 (8)	0.0213 (8)	0.0159 (7)	−0.0015 (6)	0.0058 (6)	0.0007 (6)

### Geometric parameters (Å, º)

**Table:**

S1—C2	1.6845 (16)	C10—C11	1.386 (3)
C2—N3	1.378 (2)	C11—H11	0.9500
C2—N6	1.338 (2)	C11—C12	1.395 (2)
N3—C4	1.3876 (19)	C12—H12	0.9500
N3—C7	1.4407 (19)	C13—H13A	0.9900
C4—N5	1.302 (2)	C13—H13B	0.9900
C4—C13	1.494 (2)	C13—C14	1.507 (2)
N5—N6	1.3727 (18)	C14—C15	1.367 (2)
N6—H6	0.8800	C14—C18	1.423 (2)
C7—C8	1.388 (2)	C15—H15	0.9500
C7—C12	1.385 (2)	C15—S16	1.7098 (16)
C8—H8	0.9500	S16—C17	1.7226 (16)
C8—C9	1.389 (2)	C17—H17	0.9500
C9—H9	0.9500	C17—C18	1.401 (2)
C9—C10	1.386 (3)	C18—H18	0.9500
C10—H10	0.9500		
			
N3—C2—S1	129.51 (12)	C10—C11—H11	119.8
N6—C2—S1	127.09 (12)	C10—C11—C12	120.48 (16)
N6—C2—N3	103.39 (13)	C12—C11—H11	119.8
C2—N3—C4	107.58 (13)	C7—C12—C11	118.91 (15)
C2—N3—C7	126.59 (13)	C7—C12—H12	120.5
C4—N3—C7	125.83 (13)	C11—C12—H12	120.5
N3—C4—C13	123.47 (14)	C4—C13—H13A	109.1
N5—C4—N3	111.06 (14)	C4—C13—H13B	109.1
N5—C4—C13	125.43 (14)	C4—C13—C14	112.45 (13)
C4—N5—N6	103.95 (12)	H13A—C13—H13B	107.8
C2—N6—N5	114.02 (13)	C14—C13—H13A	109.1
C2—N6—H6	123.0	C14—C13—H13B	109.1
N5—N6—H6	123.0	C15—C14—C13	123.41 (15)
C8—C7—N3	118.99 (14)	C15—C14—C18	112.18 (14)
C12—C7—N3	119.67 (14)	C18—C14—C13	124.39 (15)
C12—C7—C8	121.33 (15)	C14—C15—H15	124.1
C7—C8—H8	120.6	C14—C15—S16	111.90 (12)
C7—C8—C9	118.88 (16)	S16—C15—H15	124.1
C9—C8—H8	120.6	C15—S16—C17	93.21 (8)
C8—C9—H9	119.6	S16—C17—H17	125.3
C10—C9—C8	120.70 (16)	C18—C17—S16	109.37 (12)
C10—C9—H9	119.6	C18—C17—H17	125.3
C9—C10—H10	120.2	C14—C18—H18	123.3
C9—C10—C11	119.68 (16)	C17—C18—C14	113.35 (14)
C11—C10—H10	120.2	C17—C18—H18	123.3
			
S1—C2—N3—C4	−179.20 (12)	N6—C2—N3—C4	−0.37 (16)
S1—C2—N3—C7	−0.1 (2)	N6—C2—N3—C7	178.76 (14)
S1—C2—N6—N5	178.97 (11)	C7—N3—C4—N5	−178.60 (14)
C2—N3—C4—N5	0.55 (18)	C7—N3—C4—C13	3.6 (2)
C2—N3—C4—C13	−177.23 (15)	C7—C8—C9—C10	1.1 (3)
C2—N3—C7—C8	−63.5 (2)	C8—C7—C12—C11	−0.1 (2)
C2—N3—C7—C12	117.40 (17)	C8—C9—C10—C11	−0.2 (3)
N3—C2—N6—N5	0.10 (17)	C9—C10—C11—C12	−0.9 (3)
N3—C4—N5—N6	−0.46 (17)	C10—C11—C12—C7	1.0 (2)
N3—C4—C13—C14	−173.79 (14)	C12—C7—C8—C9	−1.0 (2)
N3—C7—C8—C9	179.97 (15)	C13—C4—N5—N6	177.26 (15)
N3—C7—C12—C11	178.94 (14)	C13—C14—C15—S16	−178.12 (12)
C4—N3—C7—C8	115.47 (17)	C13—C14—C18—C17	178.09 (15)
C4—N3—C7—C12	−63.6 (2)	C14—C15—S16—C17	−0.21 (13)
C4—N5—N6—C2	0.22 (18)	C15—C14—C18—C17	−0.4 (2)
C4—C13—C14—C15	−106.27 (18)	C15—S16—C17—C18	−0.01 (13)
C4—C13—C14—C18	75.4 (2)	S16—C17—C18—C14	0.22 (18)
N5—C4—C13—C14	8.8 (2)	C18—C14—C15—S16	0.37 (18)

### Hydrogen-bond geometry (Å, º)

Cg1 is the centroid of the C14/C15/S16/C17/C18 thiophene ring.

**Table:**

*D*—H···*A*	*D*—H	H···*A*	*D*···*A*	*D*—H···*A*
N6—H6···S1^i^	0.88	2.46	3.2866 (16)	156
C8—H8···S16^ii^	0.95	2.82	3.737 (2)	162
C10—H10···*Cg*1^iii^	0.95	2.83	3.566 (2)	135
C13—H13*B*···*Cg*1^iv^	0.99	2.76	3.409 (2)	123

Symmetry codes: (i) *x*, −*y*+3/2, *z*+1/2; (ii) *x*−1, *y*, *z*−1; (iii) *x*, *y*, *z*−1; (iv) −*x*+2, −*y*+1, −*z*+1.
